# Technology to advance infectious disease forecasting for outbreak management

**DOI:** 10.1038/s41467-019-11901-7

**Published:** 2019-09-02

**Authors:** Dylan B. George, Wendy Taylor, Jeffrey Shaman, Caitlin Rivers, Brooke Paul, Tara O’Toole, Michael A. Johansson, Lynette Hirschman, Matthew Biggerstaff, Jason Asher, Nicholas G. Reich

**Affiliations:** 1 BNext, IQT Labs, Arlington, VA USA; 2 Rockefeller Foundation, New York, NY USA; 3 Climate and Health Program, Department of Environmental Health Sciences, Mailman School of Public Health, Columbia University, New York, NY USA; 4 Center for Health Security, Johns Hopkins University, Baltimore, MD USA; 5 Taivara Ltd, Columbus, OH USA; 6 Division of Vector-Borne Diseases, Centers for Disease Control and Prevention, San Juan, PR USA; 7 The MITRE Corporation, Bedford, MA USA; 8 Influenza Division, Centers for Disease Control and Prevention, Atlanta, GA USA; 9 Leidos, Reston, VA USA; 10 Department of Biostatistics and Epidemiology, University of Massachusetts-Amherst, Amherst, MA USA

**Keywords:** Computational biology and bioinformatics, Epidemiology, Mathematics and computing

## Abstract

Forecasting is beginning to be integrated into decision-making processes for infectious disease outbreak response. We discuss how technologies could accelerate the adoption of forecasting among public health practitioners, improve epidemic management, save lives, and reduce the economic impact of outbreaks.

“*Data gaps undermine our ability to target resources, develop policies and track accountability. Without good data, we’re flying blind. If you can’t see it, you can’t solve it.*” Kofi Annan^1^

## Data, analytics are force multipliers for outbreak response

Present capacity to develop, evaluate, manufacture, distribute and administer effective medical countermeasures (e.g., vaccines, diagnostics, therapeutics) is inadequate to meet the burden of both recurrent and emerging outbreaks of infectious diseases. When such interventions are unavailable, public health measures (e.g., contact tracing, outbreak investigations, social distancing) and supportive clinical care remain the only feasible tools to slow an emerging outbreak. Decision-making under such circumstances can be greatly improved by the use of appropriate data and advanced analytics such as infectious disease modeling or machine learning. Furthermore, these analyses can guide decision-making when medical countermeasures become available, allowing them to be used in more effective ways. Data analyses already underpin public health actions such as anticipating resource requirements, refining situational awareness and monitoring control efforts^2–5^. New applications of data science and statistical analyses to disease outbreaks could provide support to decision-makers during public health crises.

Forecasting is an emerging analytical capability that has demonstrated value in recent outbreaks by informing policy and epidemic management decisions in real-time outbreak response. During the 2014–2016 Ebola virus disease (EVD) outbreak in West Africa, there was a strong push to use clinical trials to confirm that Ebola vaccines could be safe and efficacious (J. Asher, personal communication). Real-time forecasts generated during the outbreak highlighted challenges for the design of the planned clinical trials. These studies showed, based on forecasted incidence rates of EVD, that there was a strong possibility that the trials being proposed during September 2014 would not have sufficient case numbers to demonstrate significant results. This forecasting sped up discussions among senior leaders to pursue more productive, alternative trial designs (J. Asher, personal communication).

In this Comment, we discuss major limitations of the current set of tools used in forecasting outbreaks and highlight existing and emerging technologies that have the potential to significantly enhance forecasting capabilities. We focus on forecasting for outbreak management, specifically the capacity to predict short-term (i.e., days to weeks) trends of disease activity or incidence (i.e., the number and location of new cases) in an ongoing outbreak. We do not address the prediction of outbreak emergence, which is a separate endeavor with its own opportunities^6^ and challenges^7^, nor do we consider projecting multi-year trends of disease burden^8^.

**Fig. 1:**
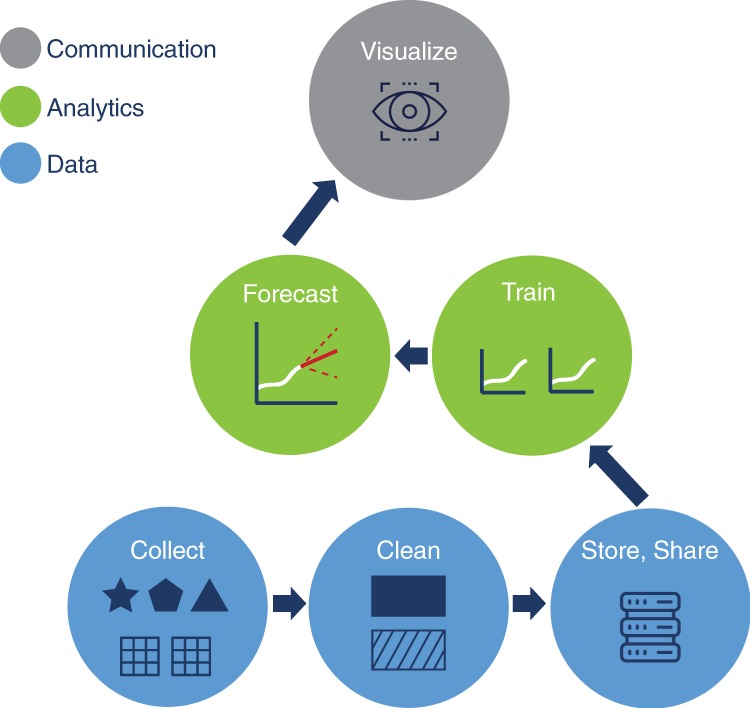
The forecasting workflow: Generating infectious disease forecasting results that will be useful for managing outbreaks follows a workflow with three main strata: data (blue circles), analytics (green circles), and communication (gray circle). Taken together, these pieces build a workflow that uses analytics to provide decision-makers with information that could be used to plan response activities

From a data science perspective, the forecasting workflow encompasses three general categories: data, analytics, and communication (Fig. 1). Each step in the process has challenges and opportunities.

## Data collection

Effective data collection and curation is essential for analytics and efficient outbreak management. Yet, for infectious disease forecasting, data quantity, quality and timeliness persist as significant challenges. Few epidemiological data are consistently reported, broadly shared, and available for decision-making during outbreak responses, especially early in outbreaks. Data collection can be a slow process, particularly in low-resource settings lacking sufficiently trained staff, with sporadic communications, limited healthcare systems, and inconsistent electrical power. Improving collection systems and advancing forecasting approaches that address these limitations and leverage existing surveillance data are necessary.

Improving diagnostic capabilities at scale should be a priority area of development. Recent advances have introduced the capacity to collect and share near real-time diagnostic results. For example, Quidel’s Sofia platform^9^ and BioFire’s FilmArray multiplex PCR^10^ both provide rapid diagnostic tests for respiratory pathogens that are wirelessly connected to cloud-enabled databases. These early examples demonstrate how rapid, aggregated, and geo-coded diagnostic test results could improve real-time tracking of population health trends. Additionally, they could enable timely and targeted clinical trial recruitment. Determining how to scale these capabilities could provide a significant source of data to improve forecasts.

## Data cleaning

Collected data is usually not in a form amenable for immediate analysis that could support decision making, and must be processed and cleaned. Data cleaning has been largely a manual, ad hoc process in outbreak forecasting efforts. Therefore, technologies to clean data would be particularly valuable for forecasting.

Technologies that translate raw, unprocessed data into structured formats would be particularly useful. For instance, software could extract data from line lists of cases or clinical notes in electronic health records, or convert data stored in non-standard formats into machine-readable data. Digitizing handwritten text reliably, quickly and securely from clinical or epidemiological records will be a persistent need for the foreseeable future.

## Data sharing

Although tools are improving, epidemiological data sharing remains a problem. Public health agencies provide data via their websites and situational reports^11,12^. These efforts are critical for supplying information to the public but the formats often cause challenges for quantitative analysts. Typically, these reports are provided with a considerable time lag, and are not machine-readable nor provided in standard formats with metadata. This impedes sharing and use of these data.

There have been instances where epidemiological data are available via informal networks of people sharing spreadsheets (D. B. George, personal communication); secure CSV file transfers^13^; or unofficial APIs^14,15^. These approaches should be lauded, but they are not long-term, enterprise solutions.

Open-science approaches to sharing data have shown promise in recent outbreaks. Epidemiologists and modelers have begun using publicly available repositories, such as GitHub, to aggregate and share digitized data in standardized formats^16–18^. This paradigm shift resulted in a rapid improvement in data-sharing capability during the 2014–2015 West Africa Ebola outbreak (D. B. George, personal communication). A team of influenza forecasters in the U.S. also has used GitHub to share forecast data to facilitate the creation of multi-model ensemble forecasts^19,20^. The shift from informal means of sharing data to robust technologies using standardized, machine-readable formats enables more rapid and meaningful engagement of a broader group of analysts. Structured open-science approaches to data sharing that are specifically tailored to forecasting applications should be further supported and explored.

## Analytics: training models

Over the past several years, academic research on infectious disease forecasting has grown and models have successfully generated predictions for pathogens such as influenza^19–21^, dengue^13^, Zika^22^, and Ebola^2^. But, scaling academic research to support public health decision-makers in real-time has received little attention and relatively scarce resources.

The U.S. Department of Health and Human Services has built models for recent outbreaks using a combination of extramural and internal analytical resources. However, the federal government and state and local public health agencies find it difficult to recruit and retain scientists capable of developing, interpreting, and communicating quantitative results. Formalized training in “outbreak science” for public health practitioners will be a vital component in ensuring that the public and private sector work-force can respond quickly in case of an emerging epidemic threat^23,24^. Even when scientists are available in public health agencies, the long and bureaucratic processes for acquiring and securing software and data technologies present significant challenges to using current and emerging data science tools.

## Analytics: forecasting

The U.S. government wisely spent decades developing weather forecasting capabilities and continues to invest in advancing the personnel, infrastructure, data, analytics and decision frameworks necessary for supporting these activities^25^. Similar efforts to develop infectious disease forecasting capabilities need to occur. To succeed, the technological architecture supporting forecasting must be evaluated in the context of ongoing outbreak response. To this end, since 2013 the U.S. Centers for Disease Control and Prevention (CDC) has fostered an open collaboration, called FluSight^4^, to improve the science and usability of epidemic forecasts of influenza for public health decision-making^21,26,27^. However, many public health agencies have limited technical expertise or capacity to adopt, advance, and modify analytical approaches and technologies *by themselves*. Maintaining progress will require sustained, collaborative work and resources from public health agencies, academia, and the private sector. Few research funding agencies provide substantial and sustained support for this type of translational work, despite a strong track record of research productivity emerging from the CDC FluSight challenge and other governmental forecasting challenges^28^. Nor have donor foundations shown leadership in this crucial area of epidemic response. If not provided with sufficient resources, public health will remain decades behind most other sectors in its use of advanced analytics.

## Visualization and communication

Forecasting results must be communicated effectively to ensure they produce actionable insights. Visualizations play a key role. Academic groups have built data visualization tools to communicate forecasts^29^, but these largely rely on customized code. Analysts who develop forecast models typically have limited time to spend on visualization and lack advanced design skills. This can lead to hard-to-understand visualizations and misinterpretation of results when used to support decision making. However, recent work by CDC has progressively refined information from forecasting results on seasonal influenza and translated that information into actionable risk communications^4^. Such efforts should be encouraged and supported.

## Conclusions

Experience from the successful application of analytical technologies across multiple industries can inform the development of technologies for infectious disease forecasting and outbreak science. Improving technologies across the forecasting workflow will significantly advance forecasting capabilities, enable involvement from multiple stakeholders (e.g., industry, government, and academia), and allow the field to develop a robust forecasting architecture. Such advances will improve public health response to outbreaks, mitigate economic losses, and save lives.
